# The British Dental Association

**Published:** 1883-10

**Authors:** E. A. Bogue


### THE BRITISH DENTAL ASSOCIATION.

We have received from Dr. Bogue the following interesting sketch of the late meeting of this Society, held at Plymouth. Accompanying the letter were newspaper reports of the proceedings and festivities, from which we shall make extracts as we find room for them.—Editor.

On the Train,
Near Plymouth, England,
August 27th, 1883.

When I was asked a few weeks ago to send a letter to the Independent Practitioner, describing any matters of interest I might run across, I little anticipated attending the meeting of the British Dental Association, but as I have done so and been both edified and delighted thereat, I send you an account, from which you can extract such items as may seem to you interesting.

Our professional brethren on this side have been more than polite, and have fairly overwhelmed me with kindnesses. I have never before been compelled to stand as a representative of my

American brethren, and it was certainly very gratifying to receive the kindly sentiments that are expressed for us.

I was delighted to meet many of our old friends, former visitors, and some of them graduates of our colleges, Dr. Waite of Liverpool, the valedictorian of his class when he graduated at the Philadelphia Dental College, being the first; we may well be proud of the compliment paid to our colleges when gentlemen like Dr. Waite are numbered among our graduates. Mr. Turner, who some years ago visited the Harvard Dental School in company with Mr. Tomes, and afterward visited several of our colleges, was also present as Honorary Secretary, in which capacity he has done an amount of work for his profession which is only surpassed by the work that he did in the interest of dental reform, work that will be appreciated by the next and the succeeding generation of dentists more than by the present one, although the present one seems to hold him in pretty high esteem, if we are to judge from the applause that arises when his name is mentioned in public.

Mr. Hutchinson, who a dozen years ago made his mark as a fine operator at the Dental Hospital in London, and who will be remembered as a visitor to New York about five years ago, read a valuable paper, deprecating the premature extraction of the temporary cuspids.

Dr. Campbell, of New Mode plates fame, was also present, and so was Dr. Bing of Paris, with some specimens of his own peculiar bridge work, beautifully carved in ivory.

Whatever may be thought of this plan of replacing lost teeth, no one can question Dr. Bing's ability, and I believe he can point to many successful operations that have been done by his own hand.

Mr. Fernald is, I believe, the only other American that was present, but he is in partnership with Capt. Rogers, a gentleman practicing our specialty at Cheltenham, so we may look upon him as half an Englishman. Among the most promising of the younger men, in a scientific way, is Mr. Storer Bennett, who for two or three years has been doing good and original work with the microscope. You may expect to hear further from him if he lives, and he will do honor to his chosen profession. Mr. Oakley Coles, whose book on deformities of the mouth will be remembered, was present.

Last and most marked of all, I will mention the President of the Association, Mr. C. Spence Bate, F. R. S., whose untiring energy seemed to pervade everything he touched, from a dinner at his own house, through all the meetings and excursions of the Society and its entertainment by the generous hosts that were provided, to the Atheneum itself, where the meetings were held, and whose prosperity as a scientific and art museum I am told is largely due to Mr. Bate's persistent and energetic and self-denying work in its behalf. I think we may congratulate ourselves, and be very proud of such a member of our profession as Mr. Bate, for the influence that he exerts for the elevation of his calling is incalculable. But I must no longer weary you with gossip, and I refer you to the enclosed accounts of the meeting for more substantial matter.

Before I leave the subject altogether, let me inform your readers that the British Association, which corresponds to our American Scientific Association, and embraces a great many of the members of the Royal Society, is to meet next year in Montreal. As several of our own profession are also members of the Association, would it not be well to extend a formal invitation to such as are to attend the meeting of the American Dental Association as our guests? Nay, more ; could we not arrange to time our meeting so as to receive these distinguished foreign visitors and make them our guests at a good meeting of the Association? They will probably come over about the first of August, and if we could invite all the English dentists who are also members of the British Association, and do so pretty soon, it might be the makeweight that would induce hesitating ones to decide in our favor, and to pay us a visit.

Yours faithfully,

E. A. Bogue.

